# Long-term outcomes of adjuvant radiation in elderly Asians with early stage IIA breast cancer after breast-conserving surgery: a population-based study

**DOI:** 10.1007/s12282-025-01810-7

**Published:** 2025-12-22

**Authors:** Shih-Min Lin, Hsiu-Ying Ku, Jun-Ping Shiau, Fang-Ming Chen, Ming-Feng Hou, Che-Yu Hsu, Tsang-Wu Liu, Hui-Ju Ch’ang

**Affiliations:** 1 Department of Radiation Oncology, Chang Gung Memorial Hospital, Taoyuan, 333423 Taiwan; 2 Graduate Institute of Clinical Medical Sciences, College of Medicine, Chang Gung University, Taoyuan, 333323 Taiwan; 3 National Institute of Cancer Research, National Health Research Institutes, 35, Keyan Road, Zhunan Town, Miaoli, 35053 Taiwan; 4 Division of Breast Oncology and Surgery, Department of Surgery, Kaohsiung Medical University, Kaohsiung, 807378 Taiwan; 5 Division of Radiation Oncology, Department of Oncology, National Taiwan University Hospital, 100225 Taipei, Taiwan

**Keywords:** Adjuvant radiotherapy, Breast cancer, Clinical practice, Low risk, Older patients

## Abstract

**Background:**

The benefit of postoperative radiotherapy (RT) for Asian older patients with early-stage breast cancer remains unclear. This study aimed to investigate the use of adjuvant RT in clinical practice to treat elderly Asians by evaluating relevant clinical factors.

**Methods:**

A total of 1,180 older adults with American Joint Committee on Cancer stage T2N0 breast cancer with tumors ≤ 3 cm were enrolled from the Taiwan Cancer Registry between January 2011 and December 2020. We used multivariable Cox proportional hazards models to control for clinical factors and propensity score matching for sensitivity analysis. Overall survival (OS) and recurrence-free survival (RFS) were estimated using the Kaplan–Meier method and the log-rank test.

**Results:**

Among the patients aged > 70 years who received hormone therapy, RFS differed significantly between those who received adjuvant RT and those who did not (hazard ratio [HR] = 0.115, *p* = 0.01). The 5-year OS rate was 93% and 76% for those with and without adjuvant RT, respectively (HR = 0.34, 95% confidence interval [CI], 0.26–0.46, *p* < 0.001). After covariate adjustment, the OS advantage from RT was significant both for patients aged 65–70 years (HR = 0.284, 95% CI, 0.155–0.520) and those aged > 70 years (HR = 0.512, 95% CI, 0.358–0.734).

**Conclusions:**

RFS was significantly improved by adjuvant RT in patients aged > 70 years with hormone-positive tumors. Furthermore, OS advantage was observed in Asian older adults with early breast cancer who received RT following conservative breast surgery. Adding RT to adjuvant systemic therapy is beneficial and well-tolerable in older Asian patients with small T2 tumors.

**Supplementary Information:**

The online version contains supplementary material available at 10.1007/s12282-025-01810-7.

## Introduction

Breast cancer is the leading type of cancer in women, with increasing incidence worldwide and in Taiwan [1, 2]. In a previous global cancer study from 1990 to 2016, Asians constitute*d* the largest proportion of patients with breast cancer worldwide [3]. The standard of care for early-stage breast cancer is breast-conserving surgery (BCS), followed by hormone therapy based on tumor characteristics [4]. Several randomized trials have shown that whole-breast radiotherapy (RT) following BCS reduces the risk of local recurrence [5–7]. However, emerging evidence suggests that RT may be omitted in Western women aged ≥ 65 years with ER-positive small tumors who are treated with breast-conserving therapy and adjuvant endocrine therapy [7–9]. Although local recurrence was more common in the group without RT at 9.5% (95% confidence interval [CI], 6.8–12.3) versus 0.9% (95% CI, 0.1–1.7) with RT, respectively, in the PRIME II trial, overall survival (OS) at 10 years was almost identical in the two groups, at 80.8% (95% CI 77.2–84.3) without RT and 80.7% (95% CI 76.9–84.3) with RT [7]. The incidence of regional recurrence and breast-cancer-specific survival did not differ substantially between the two groups [7], and the 10-year cumulative incidence of local recurrence was consistent with that in an earlier CALGB 9343 study [8]. The CALGB 9343, PRIME II, and LUMINA trials showed that adjuvant RT significantly decreased the local recurrence rate; however, it did not improve OS or distant disease-free survival [7–9]. The PRIME II trial enrolled 1,326 women aged ≥ 65 years with hormone receptor–positive, node-negative tumors ≤ 3 cm between 2003 and 2009, and earlier the CALGB 9343 trial included women with tumor < 4 cm regardless of hormone receptor status in the beginning of the trial period (1994-July 1997) [7, 8]. Consequently, the National Comprehensive Cancer Network has allowed the omission of adjuvant RT in older patients with ER-positive tumors and small tumors since 2004 [4]. However, most studies focused on Western populations [7–9], and previous studies indicate that breast cancer survival rates vary greatly between Asian and European based on different variations in treatment and genetic background [10–12]. Among Western patients eligible for the omission of post-BCS RT after the COVID-19 pandemic, the probability of receiving RT decreased by approximately one-third from 2020 to 2022 compared to 2012–2019 [13]. It is crucial to assess the treatment effects and survival in Asian women for responding to the adjuvant RT omission.

Older patients often present with medical comorbidities and a higher risk of harm related to overtreatment. Additionally, whether elderly Asian patients with early breast cancer would gain survival advantages from adjuvant RT is unclear. Large-scale cohort studies with long-term follow-up are required to evaluate the risk of overtreatment in older Asian women with early breast cancer. Therefore, this retrospective study aimed to examine the use of adjuvant RT in daily practice to treat older adults with small T2 (≤ 3 cm) and *p*N0, adjusting for clinically relevant factors.

## Patients and methods

The Taiwan Cancer Registry (TCR), a nationwide population-based cancer registry system launched in 1979, requires all hospitals in Taiwan to submit cancer data to it [14–16]. Additionally, TCR databases are linked to death certificates from the National Death Database (NDD), which provides high-quality and accurate information on the cause and date of death up to December 31, 2022. We used the TCR data from January 2011 to December 2020 to examine adjuvant therapy in women aged ≥ 65 years with tumors ≤ 3 cm, classified as stage IIA according to the 7th edition of the American Joint Committee on Cancer staging guideline.

### Recruitment criteria

**Fig. 1:**
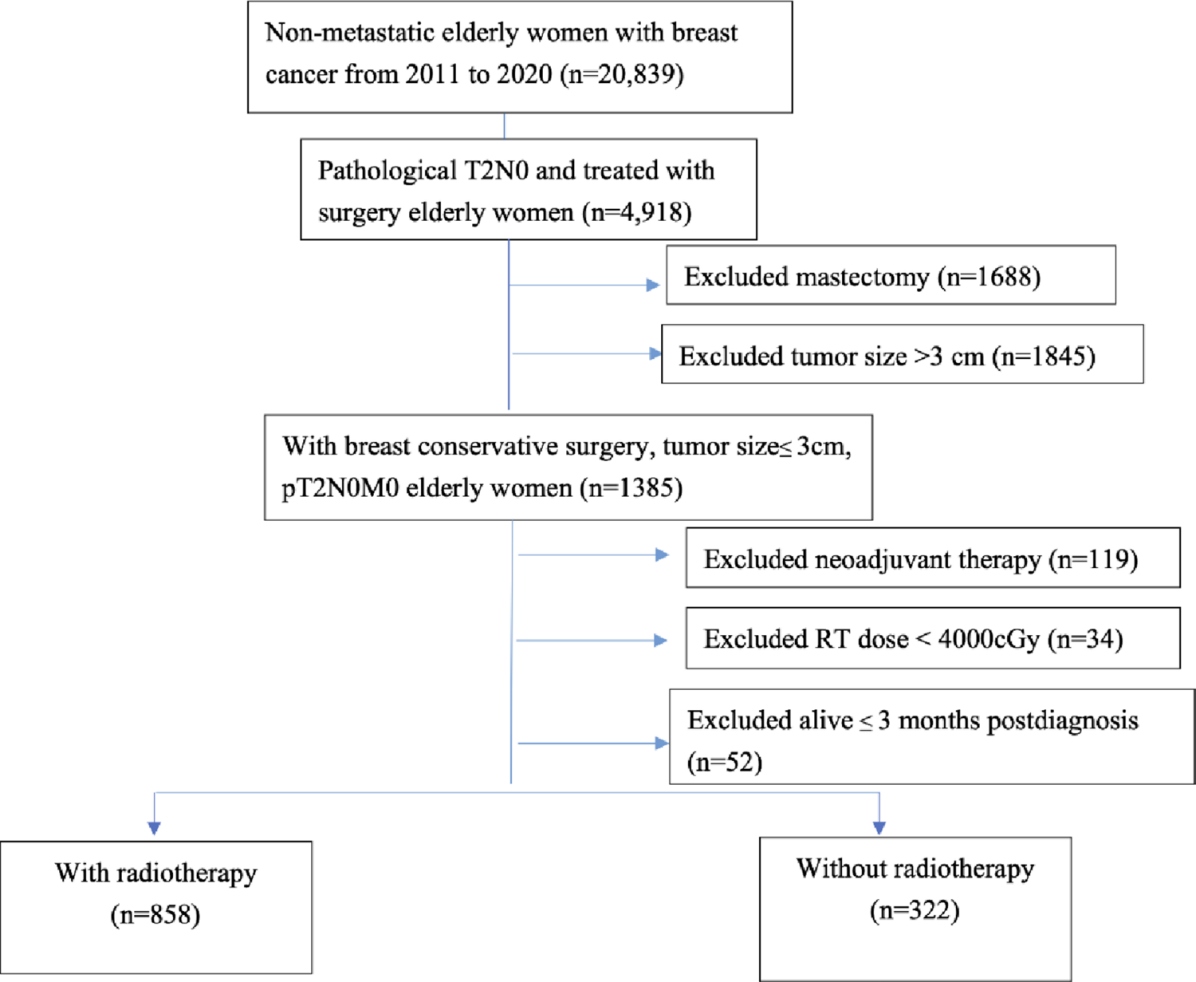
Flowchart of treatment

Figure 1 shows the research profile and identified data of age more than or equal to 65 years old female who diagnosed with non-distant metastatic disease (M0) and breast cancer between 2011 and 2020 (*n* = 20,839). To minimize the effects of treatment variability, data from patients with pathological stage T2N0 and treated with surgery were analyzed (*n* = 4,918). Patients who underwent surgical procedures coded 20–24, e.g., lumpectomy and small tumor size (≤ 3 cm) were included (*n* = 1,385). Patients who had received neoadjuvant chemotherapy or RT were excluded (*n* = 119). Those who received total cumulative radiation doses of less than 4,000 cGy were excluded from the analysis (*n* = 34). To account for surgical mortality, 52 patients who died within 3 months of surgery were excluded. Thus, this study included 1,180 patients treated with adjuvant therapy.

### End-points measurements

OS was determined from the date of the most definite surgical resection of the primary site to the date of all-cause death or last follow-up. Recurrence-free survival (RFS) was defined as the time from surgery completion to the date of distant metastasis, locoregional recurrence, death from any cause, or the last follow-up. The three patients with no information on recurrence patterns were excluded from recurrence-based data analysis. Twelve hormone receptor–positive patients who did not receive hormone therapy were excluded from the evaluation of clinical outcomes.

### Statistical analysis

Demographic and clinical characteristics differences between the non-RT (*n* = 322) and RT (*n* = 858) groups were estimated using independent t-tests and chi-square tests for continuous and categorical variables, respectively. OS and RFS rates were estimated using the Kaplan–Meier method, with differences compared using the log-rank test. All tests were two-tailed, and statistical significance was set at *p* < 0.05. Cox proportional hazard models were used to calculate the adjusted hazard ratios (aHRs) and 95% confidence intervals (CIs) to investigate the association between adjuvant therapy and survival end-points.

Sensitivity analysis was conducted using propensity score matching (PSM). For patients over 70 years with hormone receptor-positive tumors, differences in basic characteristics (age, grade, hospital level, tumor size, progesterone receptor status, Her-2-neu receptor status, adjuvant chemotherapy, and hormone therapy) between patients with and without RT were balanced using PSM. Patients with or without postoperative radiotherapy were matched 1:1 based on PS scores using a caliper width of 0.1 standard deviation. Significance was set at *p* < 0.05 for two-tailed tests and point estimates were calculated with a 95% CI. All analyses were performed using SAS version 9.3 (SAS Institute Inc., Cary, NC, USA), the R package (version 4.1.2), and SPSS (version 22.0; IBM Corp., Armonk, NY, USA).

## Results

This study included 1,180 older patients with stage T2N0 disease and small tumors (Fig. 1). In those patients receiving postoperative radiotherapy (PORT), the median radiation dose was 6000 cGy (interquartile range, 5456–6040), and the median days of RT course was 42 days (interquartile range = 35-45days). For radiotherapy treatment planning, 190 patients (22.1%) were conducted using a conventional planning technique and the remaining patients (77.9%, *n* = 668) used an intensity-modulated radiotherapy planning and/or volumetric arc modulated technique according to the Cancer Registry. Table 1 shows the demographic and tumor characteristics of the patients who received adjuvant RT (*n* = 858) and those who did not (*n* = 322). The majority (96.6%) of patients with tumors positive for ER or PR received adjuvant hormone therapy. Patients who received adjuvant RT were younger (*p* < 0.001), more likely to have received treatment at a regional hospital (*p* = 0.036), and undergone adjuvant chemotherapy (*p* < 0.001) compared to those who did not. Left breast-sided lesion (50.6% vs. 55.3%; *p* = 0.15), ER-positive status (80.4% vs. 79.5%; *p* = 0.725), HER-2-negative status (82.2% vs. 84.5%; *p* = 0.350), free tumor margin (94.4% vs. 95.0%; *p* = 0.673), tumor size (*p* = 0.311), tumor grading (*p* = 0.480), diagnosed period during this study (*p* = 0.085), were distributed equally in the patients with adjuvant RT compared to those without RT, respectively.

### Recurrence and survival

**Fig. 2:**
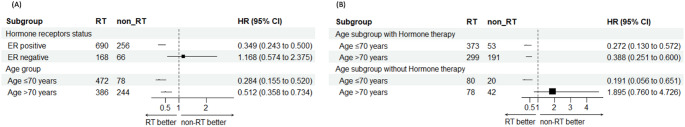
Forest plot of multivariable analysis of overall survival stratified by **A** ER status and age and **B** with or without adjuvant hormone therapy

**Fig. 3:**
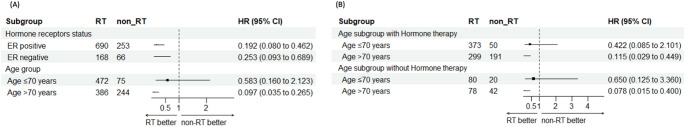
Forest plot of the multivariable analysis of recurrence-free survival stratified by **A** ER status and age and **B** with or without adjuvant hormone therapy

The median follow-up duration for 1,180 patients was 5.00 (range: 0.29–11.98) years. The 5-year OS rate was 93% in patients who received RT and 76% in those who did not. Stratifying by hormone status, the 5-year OS was 95% for patients with RT and 74% for patients without RT in patients with hormone receptor-positive tumors. For patients with hormone receptor-negative tumors, the 5-year OS was 87% with RT and 86% without RT. Table 2 presents the results of univariable and multivariable Cox regression analyses for OS. In multivariable analysis, OS was associated with receiving hormone therapy (HR = 0.39, 95% CI, 0.21–0.74), chemotherapy (HR = 0.58, 95% CI, 0.39–0.86), RT (HR = 0.45, 95% CI, 0.33–0.62), and younger age (*p* < 0.01). After adjusting for covariates, the OS advantage from RT was significant for patients aged 65–70 years (HR = 0.284, 95% CI, 0.155–0.520, Fig. 2A), those aged > 70 years (HR = 0.512, 95% CI, 0.358–0.734, Fig. 2A), those with ER-positive tumors treated with hormone therapy (Fig. 2B), and those aged 65–70 with hormone-negative tumors (HR = 0.191, 95% CI, 0.056–0.651, Fig. 2B). The 5-year RFS rate was 98% in patients who received RT and 91% in those who did not (*p* < 0.001). For patients with ER-positive tumors, the 5-year RFS was 98% and 94% for those who received adjuvant RT and those who did not, respectively (HR = 0.192, 95% CI, 0.008–0.462, *p* < 0.001). Additionally, the 5-year RFS declined from 94% to 77% in patients aged ≥ 65 years with ER-negative tumors who received and did not receive adjuvant RT (HR = 0.253, 95% CI, 0.093–0.689, *p* = 0.002). A recurrence-free benefit was observed in patients aged > 70 years with hormone-negative tumors who received adjuvant RT compared to those who did not (HR = 0.078, 95% CI, 0.015–0.400, *p* < 0.01, Fig. 3B). Among the patients aged > 70 years who received hormone therapy, RFS differed significantly between those who received adjuvant RT and those who did not (HR = 0.115, 95% CI, 0.029–0.449, *p* = 0.01; Fig. 3B).

**Fig. 4:**
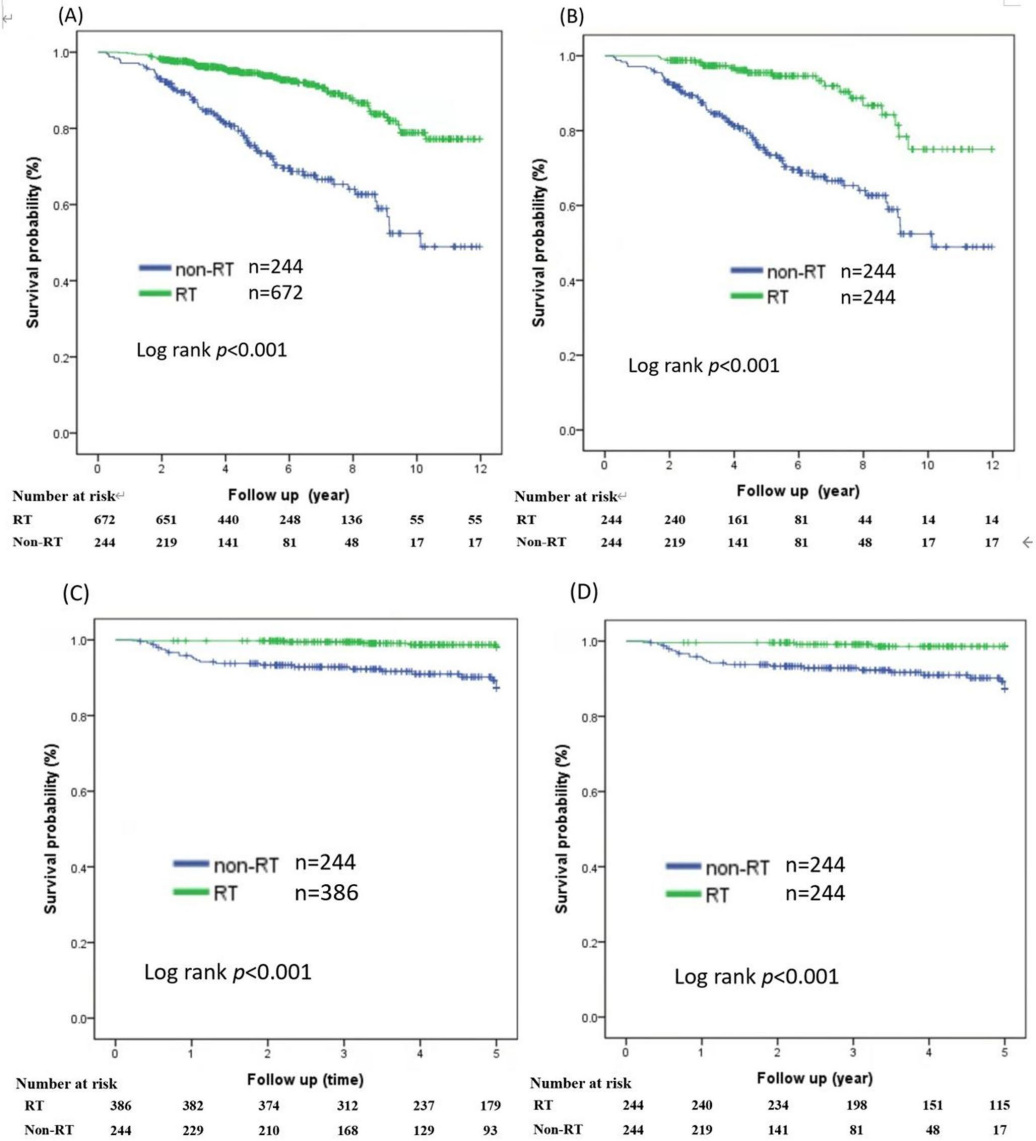
Kaplan-Meier curves for patients treated with or without adjuvant radiotherapy. **A** OS of patients who are ER-positive and received hormone therapy before PSM. HR = 0.27 (95%CI: 0.19–0.39) **B** OS of patients who are ER-positive and received hormone therapy after PSM. HR = 0.25 (95%CI: 0.15–0.42) **C** RFS of patients aged > 70 years before PSM. HR = 0.12 (95%CI: 0.05–0.32) **D** RFS in patients aged > 70 years after PSM. HR = 0.12 (95%CI: 0.04–0.38)

Sensitivity analysis with propensity score matching of OS and RFS was performed for patients stratified by hormone receptor-positive status and age (Fig. 4). A significant overall survival advantage was observed for patients with ER-positive tumors and hormone therapy who received adjuvant RT before (log-rank test, *p* < 0.001; Fig. 4A) and after PSM (log-rank test, *p* < 0.001; Fig. 4B). In patients aged > 70 years with ER-positive tumors receiving hormone therapy, the OS benefit remained significant after PSM (log-rank test, *p* < 0.01; Supplementary Fig. 1). Figure 4C, D shows the Kaplan–Meier survival curves of patients aged > 70 years who remained recurrence-free and received or did not receive adjuvant RT. Adjuvant RT significantly improved the 5-year RFS of patients aged > 70 years (log-rank test, *p* < 0.001; Fig. 4C). Furthermore, RFS remained improved with adjuvant RT in patients aged > 70 years, whereas the 5-year RFS was 99% with RT and 89% without RT after PSM (log-rank *p* < 0.001; Fig. 4D).

## Discussion

This large-scale retrospective study demonstrated a significant improvement in OS after adjuvant RT among women aged ≥ 65 years with *p*N0 and small tumor size. The implementation of adjuvant RT at an older age did not decrease during the later study period (2016–2020) or at the start of the COVID-19 pandemic in Taiwan. Adjuvant RT was administered more frequently in regional hospitals, patients aged 65–70 years, and those who received adjuvant chemotherapy. A 10-year OS benefit was observed for patients aged 65–70 and > 70 years (Fig. 2A) with small T2 breast cancer who received adjuvant RT. The 10-year OS advantages remained significant for patients aged > 70 years and those with ER-positive tumors receiving adjuvant RT after propensity-score matching. Additionally, the 5-year RFS improved significantly from 91% to 97% (Fig. 4C) for patients aged > 70 years with small T2N0 and ER-positive tumors who received adjuvant RT. For hormone-negative tumors, adjuvant RT was associated with improved OS in patients aged 65–70 years (Fig. 2B).

In our cohort of patients with hormone-positive tumors, the 10-year OS was 79% and 52% with and without adjuvant RT, respectively. Our results showed better OS than CALGB 9343, where 67% of patients received tamoxifen and radiotherapy [8]. In Taiwan, 72% of older adults with stage T2N0 and small tumors receive adjuvant RT after BCS, according to the guidelines. Conversely, a recent German study reported that 85% of older women received BCS and adjuvant RT [17]. In contrast to CALGB 9343, which mainly enrolled women aged ≥ 70 years with ER-positive tumors between 1994 and 1999, the German multicenter cohort study included 2,384 patients aged ≥ 70 years with T1–T2, node-negative disease between 2001 and 2009, shown in Table S1 [8, 17]. While adjuvant RT improved RFS in older patients with high-risk early breast cancer, no OS advantages were noted in this German study [17]. The conclusion from this German study was consistent with those of previous studies, which revealed excellent RFS but no significant OS benefit from adjuvant RT [5, 18–22]. In our study, OS advantages were noted in ER-positive tumors and patients receiving hormone therapy after PSM, as shown in Fig. 4. One possible reason might be the longer life expectancy in our cohort, with a 10-year OS of 79% compared to 67% in CALGB9343 [8]. The previous study also revealed different estimated risk of death models of breast cancer-specific survival and OS between Asian and non-Asian *populations* [23]. With long-term follow-up in our study, the survival benefit of a relatively short duration of adjuvant RT added to 5 years of hormone therapy was significant for Asian older adults. However, given that patients older than 70 years often cannot tolerate or receive adequate chemotherapy, the therapeutic landscape is considerably constrained. Subgroup analyses (Fig. 2B) indicated that postoperative radiotherapy did not significantly improve OS in hormone-negative patients aged over 70 years, yet RFS remained significantly better (Fig. 3B). These observations underscore that the availability of systemic therapy, particularly chemotherapy, might drive outcomes in patients aged more than 70 years with hormone-negative disease (Supplementary Fig. 2).

**Table 1:** Demographics and clinical characteristics for patients treated with and without radiotherapy (*n* = 1180)

Variables	Non-RT (*n* = 322)		RT group (*n* = 858)		*P* value
	n	%	n	%	
Age at diagnosis (year)					
Mean ± SD	76.24 ± 6.83		70.98 ± 5.28		< 0.001
Age at diagnosis					
65–70	78	24.2	472	55.0	< 0.001
>70	244	75.8	386	45.0	
Year of diagnosis					
2011–2015	125	38.8	287	33.4	0.085
2016–2020	197	61.2	571	66.6	
Hospital level					
Regional hospital	130	40.4	405	47.2	0.036
Medical center	192	59.6	453	52.8	
Laterality					
Right	144	44.7	424	49.4	0.150
Left	178	55.3	434	50.6	
Tumour size (mm)					
≤24	162	50.3	460	53.6	0.311
>24	160	49.7	398	46.4	
Grade					
Well differentiated	59	18.3	143	16.7	0.480
Moderately differentiated	178	55.3	479	55.8	
Poorly differentiated	79	25.4	228	26.6	
Unknown	6	1.9	8	0.9	
Margin status					
Involved	16	5.0	48	5.6	0.673
Not-involved	306	95.0	810	94.4	
ER status					
Negative	66	20.5	168	19.6	0.725
Positive	256	79.5	690	80.4	
PR status					
Negative	107	33.2	264	30.8	0.417
Positive	215	66.8	594	69.2	
Her-2-neu status					
Negative	272	84.5	705	82.2	0.350
Positive	50	15.5	153	17.8	
Hormone therapy					
Negative	74	23.0	176	20.5	0.355
Positive	248	77.0	682	79.5	
Chemotherapy					
No adjuvant	253	78.6	458	53.4	< 0.001
Adjuvant	69	21.4	400	46.6	

*SD* standard deviation;* ER* estrogen receptor;* PR* progesterone receptor;* Her-2-neu* human epidermal growth factor receptor 2

**Table 2:** Univariate and multivariate Cox proportional hazard models of overall survival

Variables	Univariate analysis					Multivariable analysis			
	5-year survival rate (%)	HR	95% confidence interval		P value	HR	95% confidence interval		P value
Radiotherapy									
No adjuvant	76	1			< 0.001	1			< 0.001
Adjuvant	93	0.34	0.26	0.46		0.45	0.33	0.62	
Age at diagnosis									
65–70	93	1			< 0.001	1			< 0.001
>70	85	2.74	1.98	3.80		1.82	1.27	2.62	
Year of diagnosis									
2011–2015	89	1			0.815	1			0.841
2016–2017	88	1.04	0.74	1.48		1.04	0.73	1.47	
Hospital level									
Medical center	87	1			0.079	1			0.033
Regional hospital	90	1.30	0.97	1.73		1.38	1.03	1.86	
Laterality									
Right	89	1			0.606	1			0.837
Left	88	1.08	0.81	1.44		0.97	0.72	1.30	
Tumour size (mm)									
≤24	90	1			0.048	1			0.186
>24	87	1.34	1.00	1.79		1.22	0.91	1.63	
Grade									
Well differentiated	90	1				1			
Moderately differentiated	88	1.24	0.80	1.94	0.340	1.35	0.86	2.13	0.189
Poorly differentiated	88	1.56	0.97	2.50	0.068	1.68	0.98	2.86	0.058
Margin status									
Involved	88	1			0.502	1			0.713
Not-involved	94	0.77	0.36	1.64		0.87	0.41	1.86	
ER status									
Negative	87	1			0.180	1			0.045
Positive	89	0.80	0.57	1.11		2.04	1.02	4.07	
PR status									
Negative	87	1			0.087	1			0.522
Positive	89	0.77	0.57	1.04		0.86	0.55	1.35	
Her-2-neu status									
1+	88	1			0.558	1			0.258
2 + or more	90	0.89	0.61	1.30		0.79	0.53	1.19	
Hormone therapy									
Negative	86	1			0.008	1			0.004
Positive	89	0.65	0.48	0.89		0.39	0.21	0.74	
Chemotherapy									
No adjuvant	86	1			< 0.001	1			0.006
Adjuvant	92	0.48	0.35	0.67		0.58	0.39	0.86	

*HR* hazard ratio ;* ER* estrogen receptor;* PR* progesterone receptor;* Her-2-neu* human epidermal growth factor receptor 2

In recent decades, concerns have been raised regarding the cardiotoxicity caused by radiotherapy [24–26]. Our study evaluated the laterality of tumors and found no significant difference in non-cancer death and any-cause death between patients with left-sided and right-sided breast cancer (Table 2). Another concern about geriatrics was their physical condition. The 10-year OS benefit was observed both for patients aged 65–70 and > 70 years (Fig. 2A) who received adjuvant RT in our study. However, the dose and frequency of adjuvant chemotherapy in patients aged > 70 years were lower than in those aged < 70 years [27, 28]. Additionally, adjuvant chemotherapy was less beneficial for RFS in patients with hormone-negative tumors aged > 70 years than in those aged 65–70 years [27]. Moreover, chemotherapy does reduce the quality of life in older women with high-risk, early breast cancer, including cognition, fatigue, physical, role, and social functioning [29]. In contrast with adjuvant chemotherapy, adjuvant RT was well-tolerable and significantly improved 5-year RFS in patients aged > 70 years, regardless of hormone status. After PSM, 5-year RFS was 99% with adjuvant RT and 89% without adjuvant RT in older patients aged > 70 years. Compared with CALGB9343, where the locoregional control rate was 90% with tamoxifen alone and 98% with tamoxifen and RT [7, 8], our study found comparable survival.

This study has some limitations that need to be considered. First, as a retrospective study, it lacked surveillance of hormone therapy compliance data and the potential bias of collected data could not be adjusted for despite using PSM. Second, performance status was only available after 2018 in the TCR database, and the patients with hormone receptor-positive breast cancer who received conservative surgery without RT were older than those who received BCS and adjuvant RT (mean age: 76.6 versus 71.0); however, age adjustment and stratification analysis were performed. Third, this study lacks records on chemotherapy duration and regimens, and the dose and frequency of standard chemotherapy are usually compromised in older adults [27, 28].

Despite these limitations, this study has several strengths. Long-term survival status was confirmed using the NDD database, ensuring reliable data for all-cause death for each patient. Additionally, this is the first large-scale, exclusively Asian population-based study to discuss the benefits of adjuvant RT in older patients.

In summary, significant differences in OS were observed in Asian older adults with early breast cancer who received RT following conservative breast surgery. Furthermore, adjuvant RT significantly improved OS in elderly patients with ER-positive tumors after PSM. Adding RT to adjuvant systemic therapy is beneficial and well-tolerable in Asian older patients with early breast cancer.

## Supplementary Information

Below is the link to the electronic supplementary material.

Supplementary Material 1

Supplementary Material 2

Supplementary Material 3

## Data Availability

The data presented in this study are available on request from the corresponding author. The data are not publicly available due to restriction of privacy and ethical policy.
