# Supplementary figures and images for: Long-term outcomes of adjuvant radiation in elderly Asians with early stage IIA breast cancer after breast-conserving surgery: a population-based study
Source: Breast Cancer. 2025 Dec 22;33(2):368–76. doi: 10.1007/s12282-025-01810-7 (PMC12960298; doi:10.1007/s12282-025-01810-7)

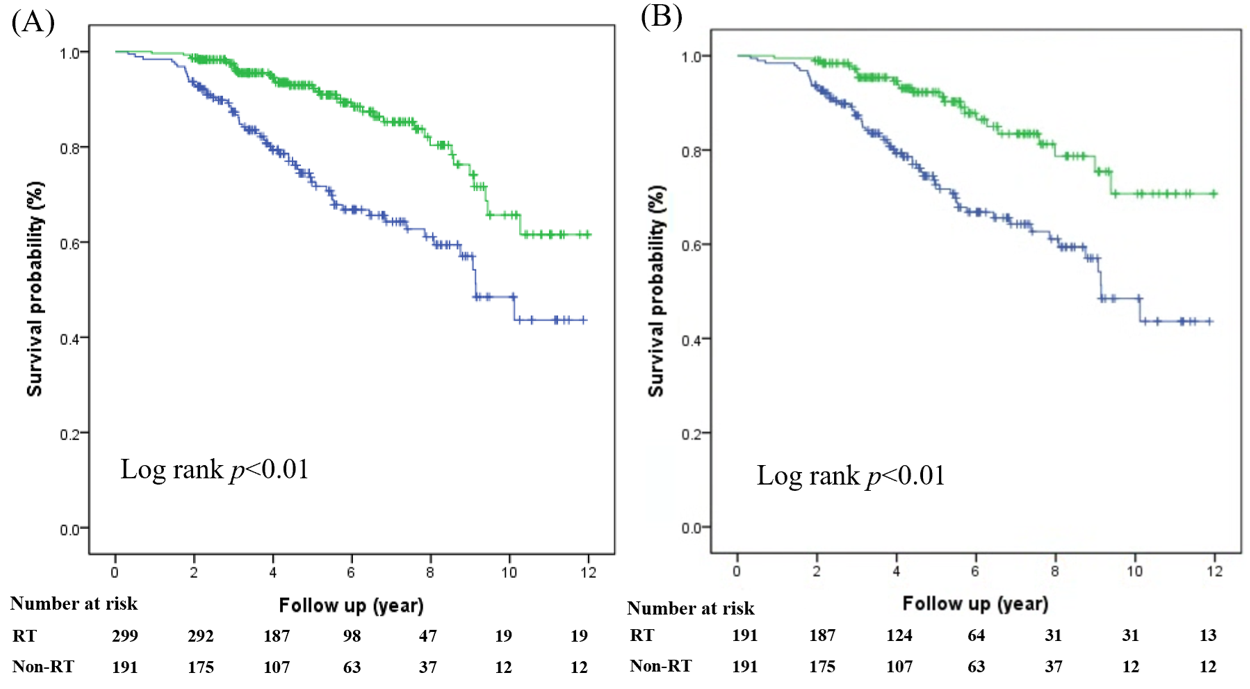

Supplement: Supplementary file 1 — Supplementary Material 1 [file 12282_2025_1810_MOESM1_ESM.tif]

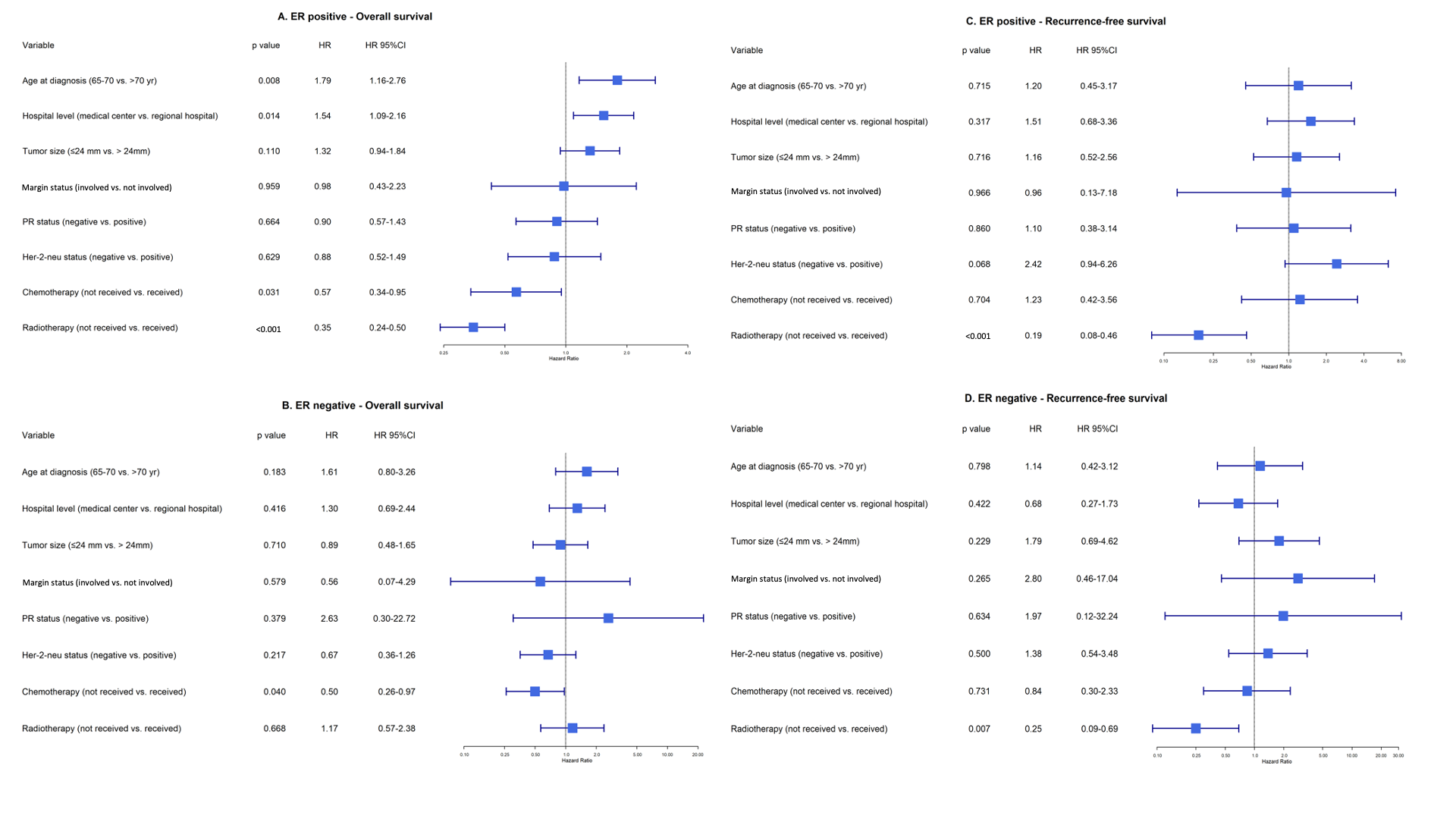

Supplement: Supplementary file 2 — Supplementary Material 2 [file 12282_2025_1810_MOESM2_ESM.tiff]
