# Supplementary material for: Long-term outcomes of adjuvant radiation in elderly Asians with early stage IIA breast cancer after breast-conserving surgery: a population-based study
Source: Breast Cancer. 2025 Dec 22;33(2):368–76. doi: 10.1007/s12282-025-01810-7 (PMC12960298; doi:10.1007/s12282-025-01810-7)
Supplement: Supplementary file 3 — Supplementary Material 3 [file 12282_2025_1810_MOESM3_ESM.docx]

Table S1. Studies Examining the Role of Irradiation After Breast-Conserving Surgery

|  | Numbers of patients | Median follow-up  (Median, years) | Enrolled  age  (years) | Tumor size | Subtype | Enrolled periods | Treatment | Overall survival (%) | Locoregional recurrence (%,) |
| --- | --- | --- | --- | --- | --- | --- | --- | --- | --- |
| CALGB 9343 | 636 | 12.6 | ≥ 70 | ≤ 4 cm | regardless of estrogen receptor status | 1994-1999 | Tamoxifen | 66 | 10(**) |
|  |  |  |  | ≤ 2 cm after Aug 1996 | ER-positive or indeterminate receptor status after August 1996 (mainly) |  | Tamoxifen and adjuvant radiotherapy | 67 | 2 |
| German multicenter study | 950 low risk  (2384 total cohort) | 3.83 | ≥ 70 | ≤ 5 cm | Low risk (Luminal A type) | 2001-2009 | With guideline adherence* | N/A | <3% |
|  |  |  |  |  |  |  | Without guideline adherence | N/A | <3% |
| Current study | 1180 | 5 | ≥ 65 | >2cm, ≤ 3cm | regardless of estrogen receptor status | 2011-2020 | With adjuvant radiotherapy | 79(**) | 1 |
|  |  |  |  |  |  |  | Without adjuvant radiotherapy | 52 | 6 (**) |

* With guideline adherence included adjuvant radiotherapy

**statistically significant
